# Adult influenza vaccination coverage before, during and after the COVID-19 pandemic in Canada

**DOI:** 10.1186/s12889-024-20854-6

**Published:** 2024-12-02

**Authors:** Ruoke Chen, Nicolas L. Gilbert, Ève Dubé

**Affiliations:** 1 Infectious Diseases and Vaccination Programs Branch, Public Health Agency of Canada, Ottawa, Canada; 2 École de santé publique de l’Université de Montréal, Montreal, Canada; 3 Département d’anthropologie, Université Laval, Québec, Canada

**Keywords:** Influenza vaccination, Impact of COVID-19 pandemic, Vaccination coverage, Place of vaccination, Public health, Canada.

## Abstract

**Background:**

Vaccination prevents seasonal influenza and its complications, particularly among high-risk populations. The COVID-19 pandemic has been reported to impact healthcare behaviors and vaccination patterns. This study aims to assess influenza vaccination coverage and changes in vaccination settings among Canadian adults from the 2018–2019 to the 2023–2024 seasons.

**Method:**

We conducted a retrospective analysis of data from multiple cycles of the Seasonal Influenza Vaccination Coverage Survey (SIVCS). Vaccination coverage was examined across different seasons, stratified by population groups. Odds ratios (ORs) were calculated to compare vaccination likelihoods across seasons, with 2018–2019 serving as the reference. Chi-square tests were applied to determine whether there were significant differences in the place of vaccination since the pre-pandemic season.

**Results:**

When comparing vaccine uptake before, during and after the COVID-19 pandemic, we observed a temporary coverage decline in 2021–2022 season (OR = 0.882, 95% CI = 0.787–0.988) compared to the pre-pandemic season in 2018–2019. By the 2022–2023 and 2023–2024 seasons, vaccination coverage returned to pre-pandemic levels. Coverage among adults aged 18–64 without chronic medical condition consistently remained lower than in other groups. The places of vaccination shifted markedly, with pharmacies becoming the predominant site, increasing from 35.4% in 2018–2019 to 57.4% in 2023–2024, while doctor’s offices saw a decline from 32.7 to 15.2% over the same period.

**Conclusion:**

Our findings highlight the transient effect of the pandemic on flu vaccine uptake in Canada. The increasing use of pharmacies for vaccinations underscores the importance of accessible and convenient vaccination sites. Future efforts should focus on maintaining and improving vaccination coverage through diverse and adaptable vaccination settings.

## Introduction

Influenza is an acute respiratory illness caused by influenza viruses. While most individuals experience symptoms such as fever, aches, and cough, certain populations, including young children, the elderly, and those with chronic medical conditions, are at increased risk of severe complications, such as secondary bacterial infections, which can significantly heighten morbidity and mortality [1]. It is ranked among the top 10 leading causes of death in Canada with an average of 12,200 hospitalizations and 3,500 deaths per years according to data from before the COVID-19 pandemic [2]. Annual vaccination is the most effective way to help prevent infection and to reduce the morbidity and mortality associated with this disease, while also helping to reduce antimicrobial resistance (AMR) spread by preventing secondary bacterial infections and decreasing the need for antimicrobial prescriptions [1, 3, 4]. Despite the proven efficacy of vaccination in mitigating the impact of influenza, coverage rates fluctuate widely across different demographics and time periods, often influenced by socioeconomic factors such as lack of health insurance and access to primary care disproportionately affect low-income and minority populations. Additionally, geographic disparities and age-related factors, particularly among older adults and those with chronic health conditions, play significant roles in influenza vaccine uptake [5, 6].

The COVID-19 pandemic has significantly altered the landscape of public health and healthcare access. The pandemic shifted focus away from routine vaccinations and lockdowns and restrictions disrupted the operation of some primary care facilities, particularly in the first months of the pandemic, potentially leading to changes in influenza vaccination behaviors and settings. Previous research has shown that pandemics can influence vaccination behaviors, For instance, during the 2009 H1N1 pandemic, changes in influenza vaccination coverage were observed as public health efforts focused on addressing the immediate threat of H1N1 [7, 8]​. Similarly, the COVID-19 pandemic redirected public health resources and attention, potentially affecting routine immunization programs, including seasonal influenza vaccination [9]. Studies have indicated a notable decline in routine vaccination rates during pandemic periods due to mitigation measures such as lockdowns and school closures [9–11]. Additionally, the shift towards remote healthcare services has raised questions about the accessibility and uptake of vaccinations [10, 12].

Understanding the impact of the COVID-19 pandemic on influenza vaccination coverage is useful for several reasons. First, it provides insights into the resilience of vaccination programs during public health crises. Second, it helps identify potential gaps and areas for improvement in maintaining high vaccination coverage during pandemics. Finally, it informs future strategies to ensure the continuity of essential immunization services in the face of public health emergencies.

The Public Health Agency of Canada (PHAC) has conducted the Seasonal Influenza Vaccination Coverage Survey (SIVCS) annually since 2015, providing valuable insights into vaccination behaviors, attitudes, and vaccination coverage across the population [13]. Adults aged 18 years and over living in Canada were surveyed regarding their influenza vaccination status, reasons for vaccination or non-vaccination, knowledge, attitudes and beliefs regarding flu vaccine and vaccination in general. These surveys assess various factors influencing vaccine uptake, including demographic characteristics, vaccination locations, and changes over time, offering a comprehensive understanding of influenza vaccination dynamics in Canada. Given the expanded role of pharmacies, which have been authorized to administer influenza vaccines in community settings, it’s important to examine changes in vaccination locations. This shift in service delivery could influence where individuals choose to get vaccinated, contributing to changes in overall vaccination behaviors and access [14].

This study aims to investigate the changes in influenza vaccination coverage across multiple influenza seasons while also examining the evolving settings where individuals receive their vaccinations. Specifically, we analyzed vaccination coverage among different population groups, including all adults aged 18 years and older, adults aged 18–64 with chronic medical conditions, adults aged 18–64 without chronic medical conditions, and adults aged 65 and older. Through this comprehensive examination, we aspire to provide valuable insights into the shifting dynamics of influenza vaccination practices, ultimately informing future public health initiatives aimed at enhancing vaccination coverage and accessibility.

## Methods

### Survey design and sampling procedures

This study utilizes data from six consecutive waves of the Seasonal Influenza Vaccination Coverage Survey (SIVCS) between 2018 and 2019 and 2023–2024. The survey is conducted annually by Léger Marketing and employs a stratified regional sampling approach to ensure national representation across all provinces and territories of Canada. In each wave, respondents were selected through random digit dialing (RDD) of both landlines and known cellphone-only household numbers, capturing the demographic that primarily uses cell phones. Interviews were conducted using Leger’s computer-assisted telephone interviewing (CATI) system, which automates the sampling process by selecting and dialing phone numbers randomly, minimizing selection bias. One adult respondent per household was interviewed, while Canadians without access to a landline or cellphone were excluded from the survey [15].

### Weighting

The survey results were weighted according to Statistics Canada’s 2016 or 2021 national census data. Leger weighted the results by age, gender, region, mother tongue, and education level to ensure that the survey results reflect the national population distribution accurately. Additionally, the weighting of respondents in the cell phone-only sample was adjusted to align with Statistics Canada’s most recent estimates of Canadian households with cellphones only, ranging from 48% for surveys conducted between 2018 and 2019 and 2022–2023, to 52% for the 2023–2024 survey. This adjustment corrects any discrepancies between the sample and the actual population proportions.

### Data collection periods and response rates

Data collection for each wave varied slightly, with the most recent collection period in 2023–2024 being longer due to an expanded sample size. Table 1 provides an overview of the data collection periods and response rates for each influenza season included in this study. Across the six waves, response rates ranged from 10 to 20%, with sample sizes spanning from 3,026 to 5,364 respondents per year.

**Table 1:** Seasonal Influenza Vaccination Coverage Survey (SIVCS) collection periods and response rates

Season	Start date	End date	Number of respondents	Response rate
**2018–2019**	2019-01-21	2019-02-24	3,737	20%
**2019–2020**	2020-01-10	2020-02-18	3,026	17%
**2020–2021**	2021-01-06	2021-02-11	3,032	16%
**2021–2022**	2022-01-04	2022-02-11	3,502	15%
**2022–2023**	2023-01-05	2023-02-20	3,558	10%
**2023–2024**	2024-01-03	2024-03-05	5,364	10%

### Statistical analysis

#### Coverage analysis

Vaccination status was determined using a standardized survey question: “From September 1st, 2023, to now, have you received the seasonal flu vaccine (also known as the flu shot)?” with response options “Yes”, “No”, and “I don’t know”. Coverage was estimated using weighted prevalence proportions, with 95% confidence interval (CI) to ensure precision and reliability of the results. Respondents who did not know their vaccination status were excluded from the analysis. Simple logistic regression was used to estimate the odds ratios (OR) for vaccination by season, comparing coverage relative to the pre-pandemic season in 2018–2019 among all adults and by population groups including adults aged 18–64 with chronic medical conditions, adults aged 18–64 without chronic medical conditions, and adults aged 65 and older.

#### Vaccination locations analysis

In addition to assessing vaccination coverage, we also analyzed changes in places of vaccination compared to the pre-pandemic season in 2018–2019. Respondents were asked the survey question: “Where did you receive the flu vaccine this time?” with options including: “Permanent or temporary vaccination Centre,” “Doctor’s Office / Health Clinic,” “CLSC / Community Health Centre or Public Health Unit,” “Hospital,” “Pharmacy,” “Workplace,” “Retirement Residence / Eldercare Centre,” and “Other.”

Using this data, we constructed contingency tables to explore the distribution of vaccination settings across influenza seasons, and prevalence rates with 95% CI were estimated. Chi-square tests were applied to compare each season to the reference season (2018–2019) for each vaccination location to determine whether there were significant changes in vaccination settings since the pre-pandemic season.

#### Exclusions

Individuals without definitive vaccination status answers, those who did not recall their place of vaccination, and those aged 18–64 who did not disclose chronic medical conditions were excluded from the stratified analysis, representing less than 1% of the study population.

All analyses were conducted using SAS Enterprise Guide 7.1.

## Results

### Overall influenza vaccination coverage

Table 2 presents influenza vaccination coverage among Canadian adults from the 2018–2019 season to the 2023–2024 season, categorized by population group. The table includes the number of respondents (n) and the weighted prevalence proportions with 95% confidence intervals (CIs).

**Table 2:** Adult influenza vaccination coverage, by population group, from 2018–2019 to 2023–2024 in Canada

Age group (years)	2018–2019		2019–2020		2020–2021		2021–2022		2022–2023		2023–2024	
	*n*	% (95%CI)	*n*	% (95%CI)	*n*	% (95%CI)	*n*	% (95%CI)	*n*	% (95%CI)	*n*	% (95%CI)
18 years and older	3726	41.8 (39.8–43.8)	3023	41.8 (39.7–43.9)	3014	40.4 (38.4–42.4)	3487	38.7 (36.9–40.6)	3535	43.5 (41.6–45.3)	5344	42.2 (40.5–44.0)
18 to 64 years without chronic medical conditions	2124	30.8 (28.2–33.4)	1558	30.0 (27.3–32.7)	1498	29.2 (26.6–31.8)	1658	26.8 (24.4–29.2)	1715	31.0 (28.6–33.4)	987	28.5 (26.1–30.8)
18 to 64 years with chronic medical conditions	770	42.8 (38.5–47.2)	668	43.6 (39.0-48.1)	646	40.5 (36.2–44.8)	713	37.6 (33.6–41.7)	583	43.1 (38.6–47.6)	583	44.1 (40.1–48.1)
65 years and older	828	69.9 (66.5–73.4)	789	70.3 (66.7–73.8)	862	70.4 (67.1–73.8)	1098	71.0 (68.1–74.0)	1198	73.7 (71.0-76.5)	2072	72.7 (70.3–75.1)

Overall, influenza vaccination coverage among all adults significantly decreased during the 2021–2022 season (38.7%) compared to pre-pandemic season in 2018–2019 (41.8%). However, the coverage increased to 43% in 2022–2023 and 42% in 2023–2024, returning to pre-pandemic levels.

Table 3 provides the odds ratios (ORs) and their 95% confidence intervals (CIs) for influenza vaccination by season and population group, with 2018–2019 as the reference season. For all adults, the odds ratio (OR) for being vaccinated in the 2021–2022 season was 0.88 (95% CI: 0.80–0.99, *p* = 0.030), indicating a substantial decline.

**Table 3:** Odds ratios for flu vaccination by season and population group, comparing each season to 2018–2019 in Canada

Season	All Adults OR (95% CI)	*P*-value	18–64 years without chronic medical conditions OR (95% CI)	*P*-value	18–64 years with chronic medical conditions OR (95% CI)	*P*-value	65 years and older OR (95% CI)	*P*-value
**2018–2019 (Reference)**	-	-	-	-	-	-	-	-
**2019–2020**	1.00 (0.89–1.13)	0.996	0.96 (0.80–1.15)	0.663	1.03 (0.80–1.33)	0.805	1.02 (0.80–1.29)	0.895
**2020–2021**	0.94 (0.84–1.06)	0.327	0.93 (0.78–1.10)	0.389	0.91 (0.71–1.17)	0.461	1.03 (0.81–1.29)	0.832
**2021–2022**	**0.88 (0.79–0.99)**	**0.030**	**0.82 (0.69–0.98)**	**0.027**	0.81 (0.63–1.03)	0.089	1.06 (0.85–1.32)	0.629
**2022–2023**	1.07 (0.96–1.20)	0.215	1.02 (0.86–1.20)	0.864	1.02 (0.79–1.31)	0.896	1.20 (0.97–1.49)	0.102
**2023–2024**	1.02 (0.91–1.14)	0.731	0.89 (0.75–1.06)	0.189	1.05 (0.83–1.34)	0.669	1.15 (0.93–1.41)	0.192

Bold values indicate significant odds ratios after adjustment at the 5% level

By the 2022–2023 and 2023–2024 seasons, vaccination coverage returned to pre-pandemic levels. The odds ratios for these seasons compared to the pre-pandemic season were not significantly different from 1, suggesting a rebound in vaccination rates. This recovery indicates that the impact of the pandemic on influenza vaccination was temporary.

### Vaccination coverage by population group

#### 18–64 years without chronic medical conditions

Among adults aged 18–64 without chronic medical conditions, the odds ratios across most seasons were similar to the 2018–2019 season, with the exception of a significant decrease in 2021–2022 (OR: 0.82, 95% CI: 0.69–0.98, *p* = 0.027), indicating a drop in vaccination coverage during this period.

#### 18–64 years with chronic medical conditions

For adults aged 18–64 with chronic medical conditions, the odds ratios remained stable, showing no significant differences across the seasons, including 2021–2022 (OR: 0.81, 95% CI: 0.63–1.03, *p* = 0.089), suggesting consistent vaccination rates over time for this age group.

#### 65 years and older

Similarly, in adults aged 65 years and older, the odds ratios were consistent across the seasons with no significant differences from the 2018–2019 reference season, with ORs ranging from 1.02 (95% CI: 0.80–1.29, *p* = 0.895) to 1.15 (95% CI: 0.93–1.41, *p* = 0.192), reflecting steady vaccination coverage among this high-risk group.

### Changes in influenza vaccination locations

Table 4 illustrates the shifts in the places where adults received influenza vaccinations from the 2018–2019 season through the 2023–2024 season.

**Table 4:** Changes in influenza vaccination locations from 2018–2019 to 2023–2024 in Canada

Season	Permenant or temporary vaccine clinic (i.e. at the mall) % (95% CI)	*P*-value	Doctor’s office / health clinic % (95% CI)	*P*-value	CLSC / Community health centre % (95% CI)	*P*-value	Hospital % (95% CI)	*P*-value	Pharmacy % (95% CI)	*P*-value	Workplace % (95% CI)	*P*-value	Retirement residence / eldercare centre % (95% CI)	*P*-value	Other % (95% CI)	*P*-value
**2018–2019 (Reference)**	4.9 (3.8-6.0)	-	32.7 (29.9–35.4)	-	7.5 (5.7–9.2)	-	5.6 (4.2–6.9)	-	35.4 (32.5–38.3)	-	7.5 (5.7–9.3)	-	1.4 (0.8–2.1)	-	5.1 (3.8–6.4)	-
**2019–2020**	4.2 (3.1–5.4)	0.417	28.2 (25.3–31.1)	0.029	5.9 (4.5–7.3)	0.162	5.2 (3.6–6.9)	0.770	40.0 (36.9–43.1)	0.033	8.4 (6.5–10.2)	0.507	1.2 (0.7–1.8)	0.669	6.8 (5.2–8.5)	0.094
**2020–2021**	6.4 (4.9–7.9)	0.114	23.0 (20.4–25.6)	< 0.001	5.8 (4.4–7.2)	0.136	3.1 (2.1–4.2)	0.007	48.6 (45.6–51.7)	< 0.001	6.7 (5.0-8.4)	0.506	1.7 (1.0-2.5)	0.560	4.6 (3.4–5.8)	0.609
**2021–2022**	9.1 (7.4–10.7)	< 0.001	22.2 (19.8–24.6)	< 0.001	3.2 (2.1–4.3)	< 0.001	2.8 (1.8–3.7)	0.001	53.4 (50.4–56.3)	< 0.001	6.7 (5.0-8.4)	0.515	1.7 (1.1–2.4)	0.528	1.0 (0.4–1.5)	< 0.001
**2022–2023**	12.1 (10.4–13.8)	< 0.001	17.4 (15.4–19.4)	< 0.001	5.2 (3.9–6.4)	0.031	2.8 (1.9–3.7)	0.001	52.3 (49.7–55.0)	< 0.001	4.6 (3.4–5.8)	0.007	0.9 (0.5–1.3)	0.153	4.6 (3.5–5.7)	0.590
**2023–2024**	12.1 (10.6–13.7)	< 0.001	15.2 (13.4–17.1)	< 0.001	3.3 (2.3–4.3)	< 0.001	2.8 (1.8–3.8)	0.001	57.4 (54.9–59.9)	< 0.001	5.0 (3.7–6.2)	0.016	1.2 (0.7–1.7)	0.587	3.0 (2.2–3.8)	0.004

### Permanent or temporary vaccine clinics

The prevalence of vaccinations at permanent or temporary vaccine clinics significantly increased from the reference season of 2018–2019 (4.9%, 95% CI: 3.8-6.0). By 2021–2022, the rate rose to 9.1% (95% CI: 7.4–10.7, *p* < 0.001), and further increased to 12.1% (95% CI: 10.6–13.7, *p* < 0.001) in the 2022–2023 and 2023–2024 seasons, respectively.

### Doctor’s office / Health clinic

The proportion of adults vaccinated in doctor’s offices or health clinics declined over the observed period. The percentage decreased from 32.7% (95% CI: 29.9–35.4) in 2018–2019 to 28.2% (95% CI: 25.3–31.1, *p* = 0.029) in 2019–2020, and continued to drop, reaching 15.2% (95% CI: 13.4–17.1, *p* < 0.001) by 2023–2024.

### CLSC / Community health centre

There was a notable decline in vaccinations at CLSCs or community health centres, from 7.5% (95% CI: 5.7–9.2) in 2018–2019 to 3.3% (95% CI: 2.3–4.3, *p* < 0.001) in 2023–2024.

### Hospital

Vaccinations administered in hospitals also saw a significant decrease, from 5.6% (95% CI: 4.2–6.9) in 2018–2019 to 2.8% (95% CI: 1.8–3.8, *p* < 0.001) in 2023–2024.

### Pharmacy

The proportion of adults vaccinated in pharmacies increased over time, rising from 35.4% (95% CI: 32.5–38.3) in 2018–2019 to 57.4% (95% CI: 54.9–59.9, *p* < 0.001) in 2023–2024, marking a significant shift toward this setting.

### Workplace

Workplace vaccinations showed some fluctuations but generally decreased from 7.5% (95% CI: 5.7–9.3) in 2018–2019 to 5.0% (95% CI: 3.7–6.2, *p* = 0.016) in 2023–2024.

### Retirement residence / Eldercare centre

The vaccination rates in retirement residences or eldercare centres remained low and did not show significant changes over time, with 1.4% (95% CI: 0.8–2.1) in 2018–2019 and 1.2% (95% CI: 0.7–1.7, *p* = 0.587) in 2023–2024.

## Discussion

### Temporary impact of the COVID-19 pandemic on influenza vaccination coverage

The results of this study indicate a significant and temporary decline in influenza vaccination coverage during the 2021–2022 season, the second year of the COVID-19 pandemic, followed by a rebound to pre-pandemic levels in subsequent seasons among all adults 18 years and older, and those aged 18–64 years without chronic medical conditions. The coverage remained stable among high-risk populations, including adults aged 18–64 with chronic medical conditions and those aged 65 and older, who are more vulnerable to influenza-related complications or hospitalization. However, vaccination coverage in these groups remained below the national target of 80%. Specifically, adults aged 18–64 with chronic medical conditions fell significantly short of this goal. While the coverage among seniors was closer to the target, it still did not reach the desired threshold. When comparing with international data, we found varied trends. In the United States, influenza vaccination coverage among adults has shown fluctuations over recent years. Data from the Behavioral Risk Factor Surveillance System (BRFSS) reveal that vaccination rates in younger adults (18–49 years) experienced a slight decline, while older age groups (50–64 years and 65 + years) showed more stable or increasing trends, particularly during and after the peak pandemic years [16]. This pattern contrasts with the Canadian experience, where overall coverage rebounded swiftly to pre-pandemic levels in 2022–2023 and 2023–2024. In Europe, findings from the Raise Awareness of Influenza Strategies in Europe (RAISE) survey indicate that influenza vaccination coverage slightly increased in most Western European countries after the first year of the COVID-19 pandemic, suggesting that the pandemic did not severely disrupt influenza vaccine uptake [17].

Several factors likely contributed to the temporary drop in influenza vaccination coverage during the 2021–2022 season. During the first year of the COVID-19 pandemic, health authorities and infectious disease experts heightened public awareness and urgency around preventing any respiratory illness, including influenza, due to the uncertainty surrounding COVID-19 and the strain on healthcare facilities [18]. However, the pandemic disrupted routine healthcare services, making it more difficult for individuals to access vaccination throughout the pandemic years due to public health interventions [12, 19–21]. The 2021–2022 SIVCS reported that 13.2% of the adult population were less likely to get the influenza vaccine due to the COVID-19 pandemic, with reasons including concerns about exposure to COVID-19 and disruptions in normal vaccination routines (e.g. lack of walk-in options) [22]. Additionally, the lockdowns and other measures may have reduced the perceived risk of contracting influenza, as both influenza and COVID-19 share similar transmission pathways. The focus on COVID-19 vaccination campaigns and mandatory policies likely overshadowed flu vaccination efforts, diverting attention and resources, and contributing to vaccine fatigue and decreased influenza vaccination rates [23–25]. The low influenza activity in the 2020–2021 season may have also reduced the perceived need for flu vaccination [26].

On the other hand, the rapid return and stabilization of influenza vaccination coverage to pre-pandemic levels indicate that the COVID-19 pandemic did not significantly increase awareness of the importance of other immunization, such as influenza vaccine. Vaccine hesitancy related to concerns about COVID-19 vaccines may have influenced attitudes towards other vaccines, including the influenza vaccine [12, 21, 27]. A recent article noted that misinformation and safety concerns about COVID-19 vaccines can spill over into general vaccine skepticism, affecting uptake of other vaccines [28]. However, some studies have shown that the pandemic led to increased intent to vaccinate against influenza, suggesting that the pandemic could be a window of opportunity to promote influenza vaccination and decrease vaccine hesitancy [12, 29, 30]. This is an area that needs further investigation to understand the differences between these reactions and how best to encourage vaccine acceptance.

For high-risk groups, such as adults with chronic medical conditions and older adults, the consistency in influenza vaccination coverage over time is likely due to the higher perceived risk of severe influenza outcomes and the prioritization of these groups by healthcare providers. However, the low uptake among younger adults with chronic medical conditions is particularly concerning given their vulnerability to severe influenza outcomes. Ensuring targeted interventions for this subgroup is crucial to improving their vaccination rates.

Despite the challenges faced during the 2021–2022 season, the return to pre-pandemic influenza vaccination levels in the 2022–2023 and 2023–2024 seasons is encouraging, although an increase in coverage might have been expected due to heightened awareness. This rebound suggests that the decline in influenza vaccine uptake was temporary and short-term, unlike the more enduring impact of the pandemic on routine childhood vaccination programs, which continue to experience declining coverage [31]. The temporary drop in influenza vaccine uptake may be associated with increased hesitancy among younger populations, influenced by pandemic-related factors. In contrast, the long-term decline in routine childhood vaccinations may due to more persistent disruptions, such as reduced access to healthcare, school closures, and shifts in healthcare priorities, which have had a cumulative effect on vaccination rates. Several factors likely contributed to the quick recovery in influenza vaccination coverage, including the co-administration of influenza and COVID-19 vaccines. The co-administration of influenza and COVID-19 vaccines provided convenient access, with 71.3% of people having received both vaccines simultaneously in 2023–2024. The overlap between the flu season and ongoing COVID-19 concerns could have prompted people to seek out additional protection against respiratory illnesses, especially given the co-administration opportunities. Expanded access through pharmacies and mass vaccination clinics originally set up for COVID-19 vaccinations also facilitated broader influenza vaccine uptake. Furthermore, British Columbia expanded its influenza vaccination program in the fall of 2021, followed by Quebec in the fall of 2022, making the influenza vaccine free for all individuals aged 6 months and older [32, 33]. These changes aligned with other Canadian jurisdictions’ policies and likely contributed to increased vaccination uptake in these provinces. These efforts, combined with public health campaigns highlighting the importance of both influenza and COVID-19 vaccines, helped restore public trust in vaccination, effectively mitigating pandemic-related disruptions and addressing vaccine fatigue [34, 35]. Subsequently, influenza vaccination coverage rebounded swiftly.

### Changes in vaccination locations

The data also shows a shift in the places where influenza vaccines were administered. Notably, there was a substantial increase in vaccinations at pharmacies, which rose from 35.4% in 2018–2019 to 57.4% in 2023–2024. Canada’s publicly funded healthcare system supports vaccination programs that differ across provinces and territories but are guided by national strategies and recommendations. This increase can be linked to the expanded role of pharmacies in Canada, where they have been authorized to administer influenza vaccines in community pharmacies [14, 36]. Studies have demonstrated that influenza vaccine uptake has modestly increased in Canadian jurisdictions where pharmacists were allowed to administer influenza vaccines given their ubiquitous distribution, extended working hours, walk-in policies and availability to people without a primary care provider [36].

Permanent or temporary vaccine clinics, such as those set up in malls, also saw increased utilization, peaking at 12.1% in 2022–2024. The increase was likely due to the mass COVID-19 vaccination campaign. These clinics offered convenient and accessible options for people to get vaccinated against COVID-19, influenza and other vaccines outside of traditional healthcare settings, which was particularly important during the pandemic to improve vaccine accessibility​ [37].

Conversely, vaccinations in doctor’s offices and health clinics declined significantly from 32.7% in 2018–2019 to 15.2% in 2023–2024. This reduction may be attributable to increased availability and convenience of alternative vaccination sites such as pharmacies and temporary clinics, as well as reduced in-person healthcare visits during the pandemic​ [12].

Overall, the shift in vaccination settings reflects a broader trend towards making vaccinations more accessible and convenient, which is crucial for maintaining high vaccination coverage, especially during public health emergencies like the COVID-19 pandemic.

### Strengths and limitations

One of the primary strengths of this study is the consistency in survey design and target population, allowing for reliable coverage and data comparison over time. The use of a stratified regional sampling approach ensures representation across all provinces and territories of Canada, making the results reflective of the entire Canadian adult population. This comprehensive sampling methodology enhances the generalizability of the findings and provides valuable insights into vaccination behaviors and trends across different demographics.

Despite the strengths of this study, several limitations must be acknowledged. First, as with any survey-based research, there is potential for response bias. Participants might overreport or underreport their vaccination status, influenced by social desirability or recall bias. Second, the exclusion of individuals without access to landlines or cellphones, although minor, could slightly bias the results, particularly among populations with lower socioeconomic status and those living in retirement residences or eldercare centers who might be underrepresented in the sample. Additionally, the response rates ranged from 10 to 20%, which is typical for telephone surveys, the reliance on quota sampling—where households unwilling to participate are replaced until quotas are met—limits the ability to accurately quantify the probability of selection. This non-probabilistic approach, coupled with the low response rates, reduces the representativeness of the sample and introduces potential nonresponse bias, as differences between respondents and non-respondents are not fully addressed.

## Conclusion

The results of this study highlight the temporary impact of the COVID-19 pandemic on influenza vaccination coverage among Canadian adults. The significant decline in vaccination rates during the 2021–2022 season underscores the disruptions caused by the pandemic to routine healthcare services and vaccination efforts. However, the rebound to pre-pandemic levels in subsequent seasons is a positive indication of the resilience of public health initiatives and the effectiveness of targeted campaigns to restore confidence in vaccination programs. The shift in vaccination locations, particularly the increased use of pharmacies and temporary clinics, highlights the importance of accessibility and convenience in maintaining high vaccination coverage. Overall, this study underscores the need for sustained public health efforts to ensure high vaccination rates, particularly during public health emergencies. Future research should focus on understanding the factors influencing vaccination behaviors in different demographic groups and developing strategies to address vaccine hesitancy.

## Data Availability

The datasets for this analysis can be accessed publicly on Library and Archives Canada, https://library-archives.canada.ca/eng.
